# Long-term follow-up in a pediatric patient with Ligneous Conjunctivitis due to *PLG* gene mutation in topical plasminogen treatment after successful use of ocular prosthesis for aesthetic rehabilitation: a case report

**DOI:** 10.1186/s13052-023-01503-x

**Published:** 2023-08-23

**Authors:** Filippo Maria Panfili, Paola Valente, Andrea Ficari, Fabiana Cortellessa, Davide Vecchio, Michaela Veronika Gonfiantini, Paola Sabrina Buonuomo, Giovanna Stefania Colafati, Emanuele Agolini, Maria Bartuli, Alessandra Claudia Modugno, Marina Macchiaiolo

**Affiliations:** 1 Academic Department of Pediatrics, Bambino Gesù Children’s Hospital, IRCCS, Rome, Italy; 2 Rare Diseases and Medical Genetics Unit, Bambino Gesù Children’s Hospital, IRCCS, Pediatric Department, Piazza Di Sant’Onofrio, 4, 00165 Rome, Italy; 3 Ophthalmology Department, Bambino Gesù Children’s Hospital, IRCCS Pediatric Hospital, Rome, Italy; 4 Department of Imaging, Neuroradiology Unit, IRCCS Bambino Gesù Children’s Hospital, Rome, Italy; 5 Laboratory of Medical Genetics Translational Cytogenomics Research Unit, Bambino Gesù Children’s Hospital, IRCCS, Rome, Italy; 6 University of Rome “La Sapienza”, Rome, Italy; 7 Ocularistica Italiana, Rome, Italy

**Keywords:** Plasminogen deficiency, Case report, Topical plasminogen, Ligneous conjunctivitis, Ocular prosthesis

## Abstract

**Background:**

Ligneous Conjunctivitis (LC) is the most common clinical manifestation of Type I Plasminogen deficiency (T1PD; OMIM# 217090), and it is characterized by the formation of pseudomembranes (due to deposition of fibrin) on the conjunctivae leading to progressive vision loss.

In past times, patients with LC were treated with surgery, topical anti-inflammatory, cytostatic agents, and systemic immunosuppressive drugs with limited results (Blood 108:3021-3026, 2006, Ophthalmology 129:955-957, 2022, Surv Ophthalmol 48:369-388, 2003, Blood 131:1301-1310, 2018). The surgery can also trigger the development of membranes, as observed in patients needing ocular prosthesis (Surv Ophthalmol 48:369-388, 2003). Treatment with topical purified plasminogen is used to prevent pseudomembranes formation (Blood 108:3021-3026, 2006, Ophthalmology 129:955-957, 2022).

**Case presentation:**

We present the case of a sixteen-year-old girl with LC with severe left eye involvement. We reported the clinical conditions of the patient before and after the use of topical plasminogen eye drops and described the treatment schedule allowing the surgical procedure for the pseudomembranes debulking and the subsequent use of ocular prosthesis for aesthetic rehabilitation.

**Conclusions:**

The patient showed a progressive response to the topical plasminogen, with a complete absence of pseudomembrane formation at a twelve-year follow-up, despite using an ocular prosthesis.

## Background

Ligneous conjunctivitis (LC) is the main complication of Type I Plasminogen Deficiency (T1PD; OMIM# 217,090), a rare autosomal recessive systemic disease caused by mutations in the gene *PLG* encoding for plasminogen (PLG; OMIM# 173350) located on chromosome 6q26, resulting in reductions in both the levels of immunoreactive PLG and its functional activity [1]. The development of ligneous fibrin-rich pseudomembranes characterizes LC. Pseudomembranes can be surgically removed, but surgery alone is ineffective since lesions usually rapidly recur [1, 2]. The surgery can also trigger the development of membranes, as well as observed in ocular prosthesis use [1].

Similarly, T1PD can affect other systems, such as the upper and lower gastrointestinal system with frequent gingival lesions (ligneous periodontitis), the respiratory system, the urinary system, female genital tracts and the central nervous system with the development of occlusive hydrocephalus [1, 2].

In the past, patients with LC were treated with surgery, topical anti-inflammatory drugs, cytostatic agents, and systemic immunosuppressive drugs with limited results [1–4]. After identifying the pathogenetic mechanism, fresh plasma was used in a few patients with positive outcomes [1, 5, 6]. In 2021 the Food and Drug Administration (FDA) approved the first plasma-derived human plasminogen drug administered intravenously to treat systemic manifestations (https://www.fda.gov/vaccines-blood-biologics/ryplazim). More recently, unlicensed topical treatment with eye drops concentrated plasminogen has been used in LC with consistent results [3, 7, 8].

A clinical trial on topic plasminogen is ongoing, but no results are available (https://clinicaltrials.gov/ct2/show/NCT04586062?cond=Ligneous+Conjunctivitis&draw=2&rank=3).

We report a rapid and persistent response to treatment with topical concentrated plasminogen despite the use of an ocular prosthesis for aesthetic rehabilitation in a sixteen-year-old girl affected by LC after T1PD caused by *PLG* gene mutation.

## Case presentation

In October 2009, a four-year-old girl was referred to the Rare Disease Unit of Bambino Gesù Children Hospital, presenting with phthisis bulbi (atrophy of the ocular bulb) of the left eye. She was the second child of Caucasian, non-consanguineous parents. Since the age of two-year-old, she developed recurrent episodes of conjunctivitis with pseudomembranes on the eyelids. After the failure of medical treatments, topical antibiotics and steroids, the lesions were surgically excised, but after a few weeks, they recurred, and conjunctivitis persisted. The surgical procedure was repeated twice.

After the third recurrence, she was referred to our unit with a suspected diagnosis of LC.

**Fig. 1:**
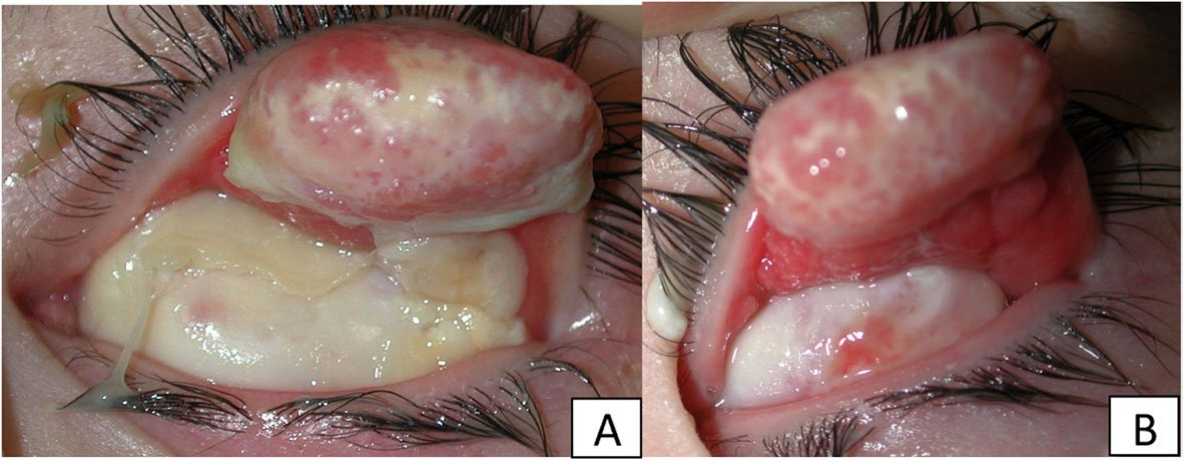
**A** and **B**, Left Eye: 1 (**A**) at presentation: Presence of a 9 mm thick pseudomembrane that involved the edge of the upper lid and the upper tarsal conjunctiva, causing ectropion of the lid 1 (**B**) after one week of intensive treatment: we observed a reduction of the whitish-yellow membranes involving the bulbar conjunctiva and fornix

**Fig. 2:**
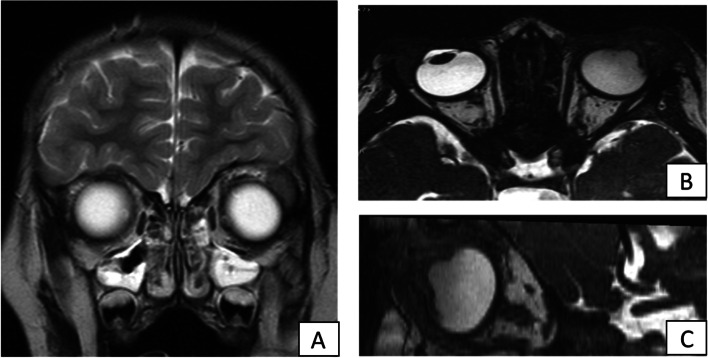
**A**, **B** and **C**: Brain MRI with Optic Nerves study, showing severe involvement of the left eye

At first examination, the left eye presented a red, woody-like pseudomembrane (9 mm thick) that involved the edge of the upper lid and the upper tarsal conjunctiva, causing ectropion of the lid (Fig. 1A). Slit lamp examination revealed a yellow-white membrane affecting the bulbar conjunctiva, fornix, and cornea, hampering the evaluation of both anterior and posterior segments. Brain MRI with Optic Nerves study, showed severe involvement of the left eye (Fig. 2A, B and C).

**Fig. 3:**
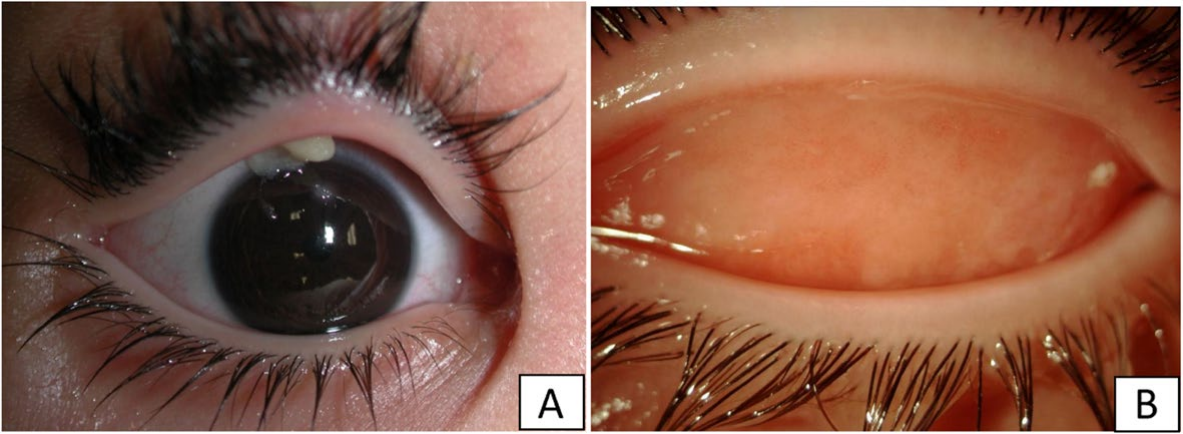
**A** and **B**, Right eye: 3 (**A**) at presentation: The right eye examination showed a small area of corneal de-epithelialization with a paracentral stromal opacity, and a whitish-yellow soft pseudomembrane involving the upper tarsal conjunctiva. The bulbar conjunctiva was not involved, and the rest of the ocular examination was normal. 3 (**B**) after one week of intensive treatment: rapid and complete response with disappearance of the pseudomembrane

The right eye examination showed a small area of corneal de-epithelialization with a paracentral stromal opacity, and a whitish-yellow soft pseudomembrane involving the upper tarsal conjunctiva. The bulbar conjunctiva was not involved, and the rest of the ocular examination was normal. (Fig. 3A).

After approval of the local Ethical Committee, treatment was started with topical plasminogen drops prepared from fresh frozen plasma (Kedrion Industrie Farmaceutiche, Lucca, Italy) in sodium hyaluronate, according to the Watts formulation [8, 9]. An intensive treatment schedule was chosen, with two drops instilled every two hours in both eyes.

A rapid and complete response was observed in the right eye after one week. **(**Fig. 3B**).** In the left eye, we observed a reduction of the pseudomembranes after one week of treatment (Fig. 1B). The therapy was further intensified three days before surgery to two drops every hour. After that the red woody-like membrane was surgically removed. The thickened subconjunctival tissue was debulked via a conjunctival approach but the ectropion and lid retraction was not corrected until the upper lid retractors were recessed. The debulked posterior surface of the tarsus was left bare to granulate and the debulked flaps of conjunctiva were approximated to the upper border of the tarsus. The eyelid margin was left intact. A prosthetic shell was inserted behind the eyelids to maintain the conjunctival sac. The plasminogen was restarted every two hours.

**Fig. 4:**
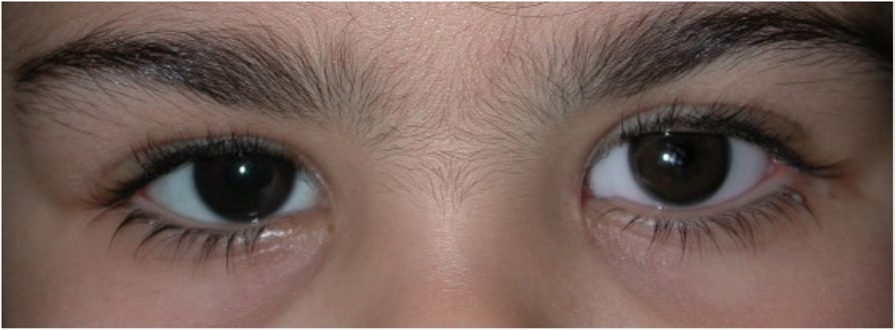
12 months after treatment

**Fig. 5:**
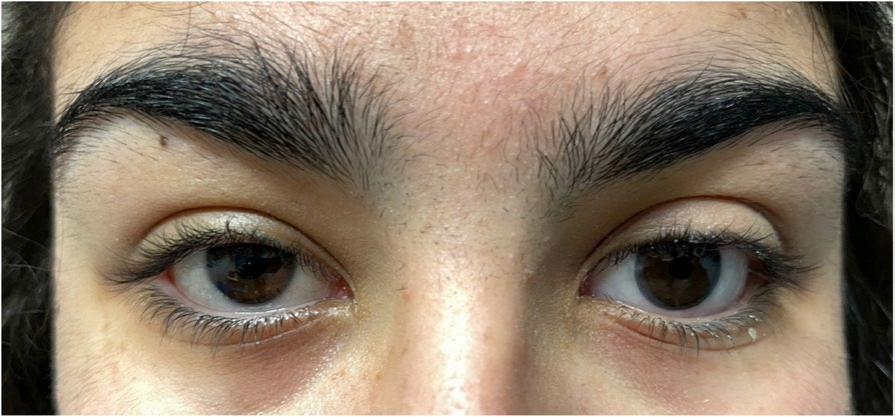
12 years after treatment

The eye drop schedule was prolonged from every two hours to every four hours, and there was no evidence of membrane reformation at the twelve-month follow-up evaluation (Fig. 4) up to the present twelve-year follow-up and the eye prosthesis is well tolerated (Fig. 5).

After three years of follow-up a nodular asymptomatic gingival hypertrophy with ulceration around the eruption site of tooth 36, was found. Non-surgical management of the lesion and strict follow-up was performed. The first molar erupted completely, with no signs of bone and periodontal pathology.

Genomic DNA was extracted from peripheral blood by using NucleoSpin tissue, according to the manufacturer's protocol (Macherey–Nagel, Germany). Whole exome sequencing (WES) was conducted on the proband and his parents by using kit Twist Custom Panel (clinical exome—Twist Bioscience) on platform NovaSeq6000 (Illumina). The bioinformatics analysis was performed trough BWA Aligner or DRAGEN Germline Pipeline systems and the sequences were aligned to reference human genome GRCh37. The DNA sequence analysis showed the variants c.112A > G (p.Lys38Glu) and c.217 T > C (p.Cys73Arg) in compound heterozygosity of *PLG* gene. The first variant was inherited by the father and the second by the mother.

## Discussion and conclusions

The most common clinical manifestation of T1PD is LC, but other systems and organs can be involved over time [1, 2, 10]. If not promptly recognized and treated, LC can evolve dramatically with severe involvement of the eyes [1, 2]. Before the treatment with topical plasminogen [3], the value of surgical treatment and use of ocular prosthesis was questioned due to the high risk of triggering episodes of recurrence [11]. Limited results were observed on the concomitant use of topical steroids or topical cyclosporine with surgery in order to decrease the local inflammation [1–4]. However, different authors in the last years have shown more stable results in patients who underwent surgery after receiving topical eye plasminogen drop instillations [7, 12].

To our knowledge, this is the first case of LC in topical plasminogen treatment after the successful implanting of ocular prosthesis. In this child was not observed the recurrence of persistent woody membranes after a 12-year long-term follow-up. Such success was likely due to the use of topical plasminogen, directly interfering in the pathogenetic pathway of the disease, interrupting the cascade responsible for the development of pseudomembrane formation. The granulation mechanism is strongly induced in non-affected populations by stimuli like surgical procedures or application of exogenous materials like the eye prosthesis [13]. Before surgery, the child was wearing an eye patch on the left eye with severe psychological consequences. Esthetic and, therefore, psychological status and quality of life for both the child and her family significantly improved after eye prosthesis insertion. The frequency of drop instillation represents an essential limitation in daily life activities. The high frequency of drop instillations can discourage adhesion to long-term therapy, thus compromising its efficacy. [3].

During the follow-up, we were able to slowly reduce the number of administrations of plasminogen drops during the day, finally trying an every four hours administration schedule.

Ligneous gingivitis represents an early clinical sign of LC oral manifestation, and it occurs in one in three patients with severe plasminogen deficiency [2, 14]. Non-surgical management and strict follow-up can be a valid option since surgical procedures seem unsuccessful [14, 15].

Plasminogen eye drops instillation can allow surgery and prosthesis insertion, with essential benefits for the patients. Further studies on larger cohorts of patients are needed to better understand the benefit of testing level of serum plasminogen, performing histological examination of pseudomembranes and the correct timing of genetic counseling. Moreover, long term observation in our patient with the relapse of symptoms after several attempts of drug reduction needing continuous modification of the treatment schedule seems to show that a discontinuation of the therapy, also after long time, is not possible. Further investigations are needed on possible different formulations to improve the therapy schedule and to prevent or treat relapse of the disease.

## Data Availability

All data have been collected from Electronic Registers. All data generated or analysed during this study are included in this published article.

## Abbreviations

LC: Ligneous Conjunctivitis
T1PD: Type I Plasminogen deficiency
